# Cartography and Epidemiological Study of Leishmaniasis Disease in Sefrou Province (2007–2010), Central North of Morocco

**DOI:** 10.1155/2020/1867651

**Published:** 2020-04-15

**Authors:** Fatima Zahra Talbi, Fatiha El Khayyat, Hajar El Omari, Saâd Maniar, Mouhcine Fadil, Amal Taroq, Abdellatif Janati Idrissi, Abdelhakim El Ouali Lalami

**Affiliations:** ^1^Laboratory Biotechnology and Preservation of Natural Resources, Faculty of Sciences Dhar El Mahraz, Sidi Mohamed Ben Abdellah University, 30000 Fez, Morocco; ^2^Regional Diagnostic Laboratory of Epidemiological and Environmental Health, Regional Health Directorate, EL Ghassani Hospital, Fez 30000, Morocco; ^3^Laboratory of Environment and Renewable Energies, University Ibn Tofail, Faculty of Sciences, BP. 133, Code 14000, Kenitra, Morocco; ^4^Natural Resources Management and Development Team, Laboratory of Health and Environment, Faculty of Sciences, Moulay Ismail University, Meknes, Morocco; ^5^Regional Health Observatory, Regional Health Directorate, EL Ghassani Hospital, 30000 Fez, Morocco; ^6^Physio-Chemical Laboratory of Inorganic Materials, Materials Science Center (MSC), Ecole Normale Supérieure, Mohammed V University, Rabat, Morocco; ^7^Laboratory of Physiology Pharmacology and Environmental Health, Department of Biology, Faculty of Sciences Dhar Mehraz, University Sidi Mohamed Ben Abdellah, 30000 Fez, Morocco; ^8^Institute of Nursing Professions and Health Techniques of Fez, Regional Health Directorate, EL Ghassani Hospital, 30000 Fez, Morocco

## Abstract

Increasing cases of leishmaniasis disease have been reported during recent years in Sefrou Province, Central North of Morocco. This study presents the epidemiological profile of the provincial population, aims at analyzing the epidemiological profile, and in particular, spatiotemporal follow-up of all cases of leishmaniasis. It is a retrospective analysis of leishmaniasis cases recorded between 2007 and 2010. The data were analyzed by SPSS software (version 20). Over a four-year period, from 2007 to 2010, there were 62 cases of leishmaniasis, 93.12% of cases of cutaneous leishmaniasis and 6.87% of visceral leishmaniasis. The case number of leishmaniasis in the Province of Sefrou varies between 0.165% and 0.0018%. For each type of leishmaniasis, the female sex was the most affected compared to the male sex. This difference cannot be considered statistically significant (*χ*2 = 0.083, *p* value = 0.77). For cutaneous leishmaniasis, all age groups were affected with a large percentage: patients aged 0–9 years with 63.11% followed by the age group [10–19] with 24.18%. Visceral leishmaniasis mainly has affected the infant population [0–9] with 83.33%. We have not observed any association between the age classes and the leishmaniasis type (*χ*2 = 6.20, *p* value = 0.4). From a spatial point of view, the majority of cases of leishmaniasis was reported in El Menzel region (67 cases) followed by Sefrou (64 cases) and Tazouta (38 cases). There is a statistically significant relationship between the type of leishmaniasis and the studied regions (*χ*2 = 52; *p* value <0.001). The study of the epidemiological profile of leishmaniasis cases may be useful in enlightening health authorities to develop screening, treatment, and control strategies to reduce the incidence rate of the disease. Other research studies can be conducted to the dynamics of the vectors of sandflies and their ecology.

## 1. Introduction

Leishmaniasis is cutaneous or visceral infection caused by an intracellular parasite of the genus *Leishmania* transmitted to humans by the bite of a sandfly [1]. They are transmitted via the female sandfly (Diptera: *Psychodidae*) of the genus *Phlebotomus* in the old world and *Lutzomyia* in the new world [2, 3]. At the global level, 350 million are at risk of acquiring the disease, and leishmaniasis causes 70,000 deaths per year [4, 5]. They are represented with an estimated annual incidence of 1.5 to 2 million new cases occurring each year [6]. In geographical terms, the CL is distributed in North Africa, West Africa, the Middle East, Central Asia, South America and Central America, and northern Bolivia in Panama [7, 8].

In the Middle East and North Africa, CL is endemic in 18 countries [9]. This region represents the highest number of CL cases in the world of which more than 100,000 cases are reported each year according to the WHO. Statements are not always notified to health authorities which say the actual burden of the disease is estimated to be three to five times higher than that reported [10]. Even if CL is not generally fatal, it has a profound impact on the social and economic life of those affected [11, 12]. In Morocco, leishmaniasis is endemic and constitutes a real health threat [13, 14]. They are classified in the list of compulsory-declared diseases according to Moroccan ministerial order *n*°683–95 from March 3, 1995. Despite these regulations, infections remain a real health problem due to the growing number of cases detected each year and the spread of cutaneous leishmaniasis [15, 16].

In terms of incidence, cases have become very frequent; the number of patients continues to increase, geographical distribution spreads, and new endemic foci. Several risk factors have led to the emergence of new outbreaks of leishmaniasis in Morocco and the increase in the number of cases [17–19]. Human cutaneous leishmaniasis (CL) is caused by three major species: *Leishmania major*, *L*. *tropica*, and *L*. *infantum* [20]. The epidemiological situation of leishmaniasis in Morocco and the distribution of *Leishmania* species vary from one region to another. According to the literature, many studies have shown that cases of CL in south-eastern Morocco were mainly caused by *L*. *major* with presence of the *L*. *tropica* case also [21, 22]. In central Morocco, *L*. *tropica* is the only agent responsible for CL cases in certain provinces [23]. In others, this species has been isolated alongside a few cases of *L*. *infantum* [24, 25]. However, at Southwest, *L*. *tropica* is the only prominent species [13, 26]. In the North, limited cases of *L*. *infantum* have also been isolated with the presence of major cases of *L*. *tropica* [27]. In northern Morocco, a study in the Tangier Tetouan-Al Hoceima region showed that two *Leishmania* species (*L*. *infantum* and *L*. *tropica*) are presented in the northern region of Morocco with a predominance of *L*. *infantum* [28]. In our region, the Fes-Boulemane region recorded 2,843 cases compared to the total of 42,491 cases recorded in Morocco during the period from 2000 to 2011 [29]. Data used are adopted for the epidemiological surveillance inserted in the register of the Medical Delegation of the Sefrou Province. The purpose of this retrospective study, from 2007 to 2010, aims at analyzing the epidemiological profile and, in particular, spatiotemporal follow-up of all cases of leishmaniasis recorded by the Medical Delegation of the Province of Sefrou.

## 2. Materials and Methods

### 2.1. Study Area

Sefrou Province covers an area of 3,520 km^2^. It belongs to the region of Fes-Meknes. It is located in northern central Atlas and medium, and it is limited to the east by the Province of Taza, in the west by the province of El Hajeb, in the north by Fes prefectures, and the south by the provinces of Ifrane and Boulemane. Most of these mountainous provinces are endemic and considered as focus of human CL [20, 22, 25]. The maximum and minimum mean monthly temperatures were, respectively, 25°C (July) and 10°C (December). The precipitation varied from 460 mm to 642 mm. The vegetation is characterized by very important cultivated vegetation. In 2011, the total population of this province was estimated by 260,000 inhabitants [30]. Fifty-two percent of the population resides in the rural zone. Agriculture and tourism are the most important sectors in the economy of this province.

### 2.2. Data Analysis and Mapping

The data were collected from registers of the Medical Delegation of the Sefrou Province. All cases of CL recorded on the survey forms were confirmed by parasitological diagnosis direct. CL patients with clinical lesions of CL were passively received during the study period at the local laboratory of the health center of Sefrou Province. A direct microscopic examination (*G* ✕ 1000) was used for the detection of *Leishmania* amastigotes, which indicates a positive skin lesion, and the corresponding slides are received at the National Reference Laboratory for Leishmaniasis in Rabat for control and confirmation. The studied period was four years between 2007 and 2010. The number of reported cases was grouped according to the reporting year and by location (municipalities and sectors) in Microsoft Excel tables on which calculated the total number of population and the number of reported cases.

The descriptive analysis has included the age, the sex, and described the evolution of the cases in the time and space. Statistical analysis was performed by SPSS software (version 20). The *χ*2 test was used for comparison of categorical variables. For all tests, the significance level was 0.05.

We studied the distribution of CL and VL cases using Qgis 2.18 software by integrating leishmaniasis disease data into the geographic information system.

## 3. Results and Discussion

### 3.1. Distribution of Leishmaniasis Cases according to Age

**Figure 1:**
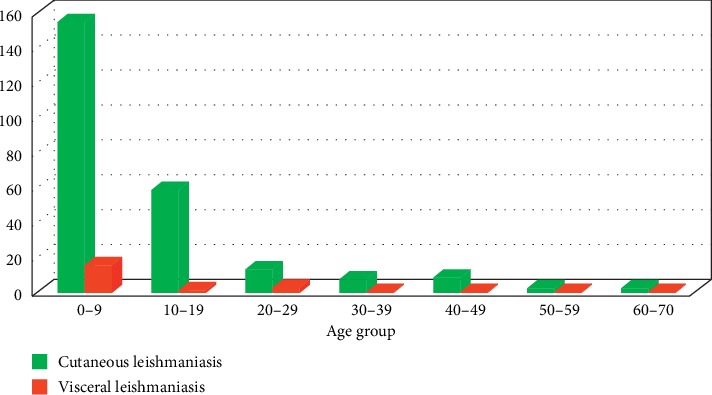
Distribution of cases of CL and VL with the age group (2007–2010).

Figure 1 shows the distribution of cases of leishmaniasis according to the age classes of the patients. We have noticed that leishmaniasis spares no age group for both types of leishmaniasis.

For CL, it was observed that all age groups were affected by this condition, but the one from 0 to 9 years was the most reported with a percentage of 63.11% followed by the age group ranging from 10 to 19 years with 24.18%. The ages ranging from 20 to 29, 30 to 39, and 40 to 49 were also affected but at low percentages with, respectively, 4.91%, 2.86%, and 3.27%. The distribution of cutaneous leishmaniasis according to the age classes showed that no age group is spared by CL with a predominance of the infantile population. A percentage of 63.11% was reported for persons aged from 0 to 9 years, which is consistent with that obtained in previous publications [2, 31]. The studies carried out at Azilal (Arroub et al., 2012) has also showed that the age range from 0 to 9 years is most affected by CL (72.4%).

For VL, it was noted that this pathology has mainly affected the infant population from 0 to 9 years with a percentage of 83.33%. Patients aged 10 to 29 years accounted for a low percentage of 16.66%. We did not observe any association between the age classes and the leishmaniasis type (χ2 = 6.20, *p* value = 0.4). Visceral leishmaniasis has been reported with minor cases for the adult age group, bearing in mind that this type of leishmaniasis is mainly the prerogative of the child. This result also corroborates with the work of Zougaghi et al. [32]. This is probably due to the frequency of visits to the school environment (active screening), as well as the lack of immunity in this age group to leishmaniasis.

### 3.2. Distribution of Leishmaniasis Cases according to Gender

The distribution of CL and VL cases according to sex is shown in Table 1. The affected population consisted of 154 females (58.77%) and 108 males (41.22%). The percentages between girls and boys differs significantly (*χ*2 = 8.07, *p* value = 0.004). Neither the masculine nor the feminine gender of this pathology of human leishmaniasis has been spared.

Depending on each type of leishmaniasis, we found a predominance of the female sex compared to the male one for both forms of cutaneous and visceral leishmaniasis. However, this difference cannot be considered statistically significant (*χ*2 = 0.083, *p* value = 0.77).

The analysis of the results of the distribution of cases of leishmaniasis according to sex showed that both sexes were affected by this pathology. The sex ratio is in favor of women as has already been mentioned by some authors [2, 31]. Analysis of the number of positive cases in Sefrou Province from 2007 to 2010 showed that cutaneous leishmaniasis affects women more than men with 59.01% and 40.98%, respectively, with one sex ratio of 0.69. This result is in agreement with that reported in other Moroccan foci, notably in Tadla-Azilal focus, both genders are concerned by CL; females were more affected (53% of total cases) [33]. This could mainly be attributed to the fact that women, for aesthetic reasons, consult the health centers more frequently than men. Note that it is the face that is most often affected by the disease. In addition, girls take care of domestic activities and thus remain in prolonged contact with the endophilic species or vectors of leishmaniasis. Men, due to their occupation and/or negligence of painless skin lesions, rarely consult or only in case of complications. Furthermore, our result is consistent with that of Guessouss et al. [34] in the Nordic provinces of Morocco, the results of which show that the female sex is mostly affected by CL with a rate of 56%. At the level of Chichaoua, the sex ratio was observed at the order of 0.8 [35]. For VL, the distribution was marked by a sex ratio of 0.8 for females in the Province of Sefrou. In Algeria, some authors have also reported a sex ratio greater than one at the order of 1.53 [36].

### 3.3. Temporal Distribution of Leishmaniasis Cases

#### 3.3.1. Annual Distribution of Leishmaniasis Cases

**Figure 2:**
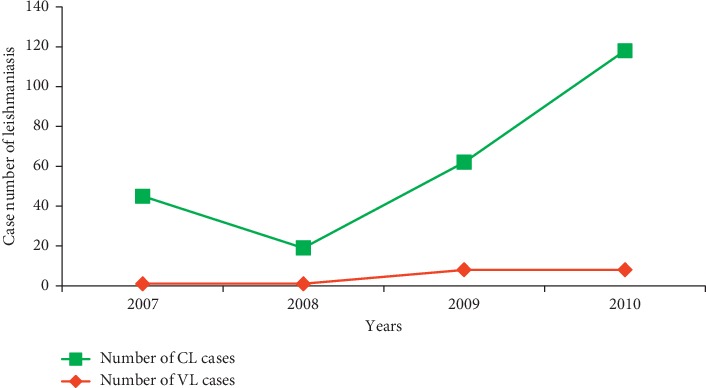
Evolution of the number of cases of leishmaniasis in Sefrou Province during 2007–2010.

Figure 2 shows the evolution of the number of cases reported in the period from 2007 to 2010. In general, leishmaniasis is still present in the Province. The minimum number of cases of leishmaniasis was recorded in 2008 with 20 cases, and the maximum number of cases was recorded in 2009 and 2010 with 70 and 126 cases, respectively.

From 2007 to 2008, we noticed a decrease in the number of cases (26 cases), and in the period between 2008 and 2010, the number of cases of cutaneous leishmaniasis increased exponentially, from 19 cases in 2008 to 118 cases in 2010 (Figure 2). The registration of VL cases revealed a census of a single case reported in 2007 and 2008 and of 8 cases in 2009 and 2010 (Figure 2).

These results corroborate with those obtained at the national level during the same period, from 4,854 to 8,846 cases [29]. This could be explained by the implementation of the strategy of the fight against this disease and the companions of information led by the Ministry of Health according to the national plan of the fight against leishmaniasis launched in 2009 (Integrated Management of Vector Control (IMVC) named GILAV in French (G: Gestion, I: Intégrée, L: Lutte, A: Anti, V: Vectorielle). The two main objectives of the program: stop the transmission of leishmaniasis in all active foci and avoid the spread of leishmaniasis to other risk areas by preventive measures. This program was based on public awareness and the intensification of mass screening activities in schools and at risk communities following the recommendations of the response plan. These climbs triggered the implementation of a national response plan to stop the increase in 2010. The response plan was followed by other strategies that aim to make the National Leishmaniasis Control Program a success: the creation of a national research committee on leishmaniasis to evaluate the WHO guide to follow-up of leishmaniasis “Leish-guide,” evaluation of the National Program to Combat Leishmaniasis, and launch of the IMVC performance action plan, this time specific to leishmaniasis, and the organization of training sessions for managers as well as for microscopists [37]. In addition, Morocco has adopted Integrated Management of Vector Control (IMVC) since 2015 as a strategy for implementing vector control. This strategy initiated by the WHO was institutionalized in 2014 by a joint decision on the IMVC which was signed by the Ministers of Health, the Interior, Agriculture, and the Environment (intra- and intersectoral collaboration: intrasectoral collaboration, intersectoral collaboration, and international collaboration).

#### 3.3.2. Monthly Distribution of Leishmaniasis Cases

The monthly evolution of the cases of the CL made it possible to observe two peaks; the first is relatively the most important in May with 47 cases and the second in the month of December with 39 cases. This could be related to the seasonal dynamics of vector populations in these areas, or this is mainly due to the latent phase between infection and the onset of clinical symptoms to perform the diagnosis.

**Figure 3:**
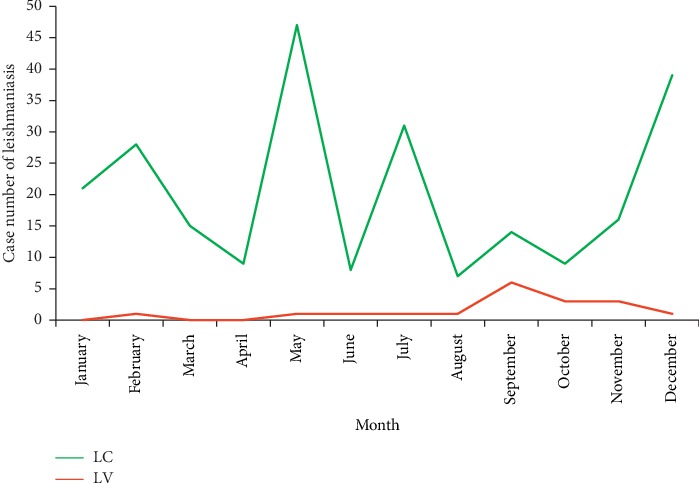
Monthly distribution of leishmaniasis cases in Sefrou Province during 2007–2010.

Also, we found a gradual increase in cases of VL, ranging from 1 case in February to 6 cases in September. However, this increase was observed between August and September (1 to 6 cases). We also noted a fall in the number of cases of VL ranging from 6 in the month of September to 3 in the month of October (Figure 3).

However, this decrease was noted important between the months of November and December (from 3 to 1 case). In addition, our observations are in line with the typical aspect of the transmission of this type of leishmaniasis (CL), whose infestation phase dates back to the summer-autumn period of the past year, which is closely related to the dynamics of the vector *Phlebotomine*, *Ph*. *sergenti* [38–40].

According to the Khi-2 test, there is a significant relationship between the percentage of leishmaniasis cases and months (*χ*2 = 33.36, *p* value <0.001).

### 3.4. Spatial Distribution of Leishmaniasis

The distribution of leishmaniasis cases in the Province of Sefrou from 2007 to 2010 over time varies from one commune to another. For the municipalities of Tazouta, Azzaba, and Sefrou, the number of cases has evolved gradually over time (Table 2).

In 2007, the spatiotemporal distribution of leishmaniasis cases marked a predominance in El Menzel (19 cases) followed by Ahl Sidi Lahcen (6 cases) and Bir Tam Tam (5 cases). The other cities are low proportions, and some are considered healthy towns without any reported case. Between 2007 and 2008, we observed a decrease in the number of patients, but in 2008, a new outbreak appeared in that of the municipality of Adrej with only one case. In 2009, the appearance of another outbreak was in that of Ain Cheggeg (1 case). For Ighazrane and Ouled Mkoudou, no case was recorded until 2010. During this year, the epidemiological situation of leishmaniasis is characterized by another image, and the urban commune of Sefrou took the first rank (37 cases) followed by that of the rural commune Tazouta (23 cases) in the second position and El Menzel with 16 cases (Table 2).

**Figure 4:**
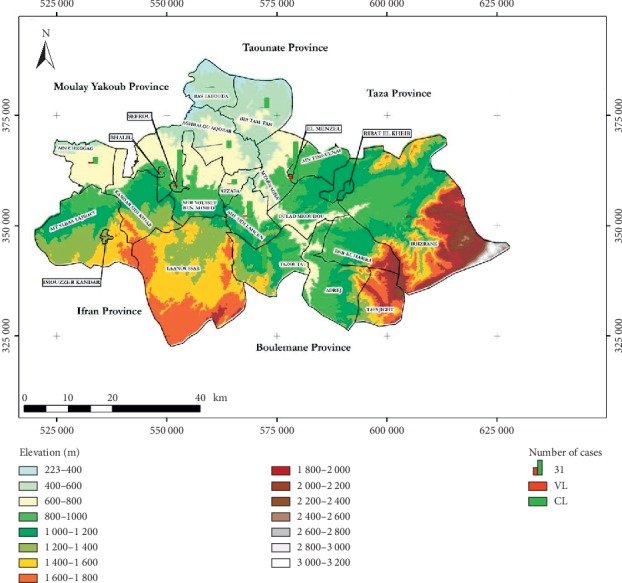
Distribution of leishmaniasis cases by communes, Sefrou Province, during 2007–2010.

From a topographic point of view, the relief has been shown to be an important ecological factor. In addition, Province of Sefrou is situated in Middle Atlas Mountains, Central of North Morocco, and considered an endemic area of human leishmaniasis. Three zones were highly prevalent for CL (Figure 4): El Menzel, Sefrou, and Tazouta. The maximum mean number of CL was situated in altitude interval between 800 and 1,000 m (Figure 4).

Among the 18 cases of VL reported in the Province of Sefrou, the municipality of El Menzel recorded five cases of VL (22.77%) followed by the commune of Sefrou which is characterized by the presence of four cases (22.22%).

Moreover, we note that there is a statistically significant difference between the percentages recorded for each commune (*χ*2 = 352.9, *p* value <0.001). In addition, we have also observed that the percentages of the two cases of leishmaniasis differ from one commune to another, showing that there is a statistically significant relationship between the type of leishmaniasis and the communes studied (*χ*2 = 52; *p* value <0.001).

Sefrou Province seems to be a typical home of CL like the other Moroccan regions [38]. *L*. *tropica* and *L*. *infantum* are the confirmed parasite species responsible for this area [25]. Our study area belongs to the semiarid located at an altitude between 220 and 2,000 m. Indeed, Rioux et al. in 1984 showed that the distribution of various vector species of leishmaniasis is mainly linked to the climate [41]. At the level of the study area, the result could be explained by the type of climate prevailing in this region, the semiarid microclimate and the mountainous areas in which *Phlebotomus sergenti* was the most commonly collected sandfly species in the same area followed by *Phlebotomus perniciosus* and *Phlebotomus longicuspis* [38]. *Ph*. *Sergenti* is a species known as a vector of *L*. *tropica* in Morocco, which was identified at Tanant by Guilvard et al. [42]. Different studies have shown the existence of this species at different bioclimatic stages but especially in semiarid and in a range of altitude varying from 800 to 1,000 m [38, 43–45]. This corroborates the distribution of CL cases at altitude concentrating in the same study area. The occurrence of leishmaniasis is linked to several risk factors [17], as well as environmental bioecological factors and climate change [46]. The areas affected by these plagues are characterized by the presence of slaughterhouses, the accumulation of livestock waste, stables, caves, and without forgetting the presence of environmental conditions favorable to the biological development of sandfly vectors [47].

## 4. Conclusion

The retrospective study of the Province of Sefrou has shown that the number of cases continues to increase. Efforts to combat this disease require continuous monitoring following awareness and information campaigns for populations at risk and to encourage and monitor scientific studies in this area. This work is very important to follow work related to the socioeconomic factors impacting the spatiotemporal dynamics of the disease as well as the factors favoring the distribution of sandfly vectors of leishmaniasis and impacting their proliferation in order to minimize the risk of transmission and dispersion leishmaniasis in endemic foci.

## Figures and Tables

**Table 1:** Distribution of leishmaniasis by sex.

Type of leishmaniasis		Sex		Total
		Male	Female	
Cutaneous leishmaniasis	Effective	100	144	244
	Percentage of CL (%)	40.98	59.01	100

Visceral leishmaniasis	Effective	8	10	18
	Percentage of VL (%)	44.44	55.55	100

**Table 2:** Representation of the spatiotemporal dynamics of cases of leishmaniasis (2007–2010).

Commune	2007		2008		2009		2010	
	CL	VL	CL	VL	CL	VL	CL	VL
Adrej	0	0	1	0	0	0	0	0
Aghbalou Aqourar	1	0	1	0	5	0	5	0
Ahl Sidi Lahcen	6	0	5	0	4	1	17	1
Ain Cheggag	0	0	0	0	1	1	9	1
Azzaba	1	0	1	0	1	0	4	1
Bir Tam Tam	5	0	4	0	4	0	3	0
Ighazrane	0	0	0	0	0	0	0	2
Ouled Mkoudou	0	0	0	0	0	0	4	0
Rass Tabouda	0	0	0	0	0	0	0	0
Tazouta	4	0	4	0	7	0	23	0
Bhalil	3	0	0	1	2	1	1	0
Imouzzer Kandar	0	0	0	0	0	0	0	0
El Menzel	19	1	3	0	24	3	16	1
Sefrou	5	0	5	0	14	2	37	1
Ribat El Kheir	0	0	0	0	0	0	0	0
Zaouiat Bougrine	0	0	0	0	0	0	0	0
Ain Timguenai	0	0	0	0	0	0	0	0
Dar El Hamra	0	0	0	0	0	0	0	0
Mtarnagha	0	0	0	0	0	0	0	0
Tafajight	0	0	0	0	0	0	0	0
Ait Sebaa Lajrouf	0	0	0	0	0	0	0	0
Kandar Sidi Khiar	0	0	0	0	0	0	0	0
Laanoussar	0	0	0	0	0	0	0	0
Sidi Youssef Ben Ahmed	0	0	0	0	0	0	0	0
Immouzer Kandar	0	0	0	0	0	0	1	0

## Data Availability

No data were used to support this study.
